# RDGN-based predictive model for the prognosis of breast cancer

**DOI:** 10.1186/s40164-020-00169-z

**Published:** 2020-06-15

**Authors:** Bing Dong, Ming Yi, Suxia Luo, Anping Li, Kongming Wu

**Affiliations:** 1 Department of Molecular Pathology, The Affiliated Cancer Hospital of Zhengzhou University & Henan Cancer Hospital, Zhengzhou, 450008 China; 2 Department of Oncology, Tongji Hospital of Tongji Medical College, Huazhong University of Science and Technology, Wuhan, 430030 China; 3 Department of Medical Oncology, The Affiliated Cancer Hospital of Zhengzhou University & Henan Cancer Hospital, Zhengzhou, 450008 China

**Keywords:** Breast cancer, RDGN, DACH, EYA, SIX, Predictive model

## Abstract

**Background:**

Breast cancer is the most diagnosed malignancy in females in the United States. The members of retinal determination gene network (RDGN) including DACH, EYA, as well as SIX families participate in the proliferation, apoptosis, and metastasis of multiple tumors including breast cancer. A comprehensive predictive model of RDGN might be helpful to herald the prognosis of breast cancer patients.

**Methods:**

In this study, the Gene Expression Ominibus (GEO) and Gene Set Expression Analysis (GSEA) algorithm were used to investigate the effect of RDGN members on downstream signaling pathways. Besides, based on The Cancer Genome Atlas (TCGA) database, we explored the expression patterns of RDGN members in tumors, normal tissues, and different breast cancer subtypes. Moreover, we estimated the relationship between RDGN members and the outcomes of breast cancer patients. Lastly, we constructed a RDGN-based predictive model by Cox proportional hazard regression and verified the model in two separate GEO datasets.

**Results:**

The results of GSEA showed that the expression of DACH1 was negatively correlated with cell cycle and DNA replication pathways. On the contrary, the levels of EYA2 and SIX1 were significantly positively correlated with DNA replication, mTOR, and Wnt pathways. Further investigation in TCGA database indicated that DACH1 expression was lower in breast cancers especially basal-like subtype. In the meanwhile, SIX1 was remarkably upregulated in breast cancers while EYA2 level was increased in Basal-like and Her-2 enriched subtypes. Survival analyses demonstrated that DACH1 was a favorable factor while EYA2 and SIX1 were risk factors for breast cancer patients. Given the results of Cox proportional hazard regression analysis, two members of RDGN were involved in the present predictive model and patients with high model index had poorer outcomes.

**Conclusion:**

This study showed that aberrant RDGN expression was an unfavorable factor for breast cancer. This RDGN-based comprehensively framework was meaningful for predicting the prognosis of breast cancer patients.

## Introduction

In the United States, breast cancer is the most commonly diagnosed cancer and second leading cause of cancer-related death in women [1, 2]. According to the presence or absence of molecular biomarkers, breast cancer could be categorized into three major subtypes: Luminal (hormone receptor positive and Her-2 negative), Her-2-enriched (Her-2 amplified), Basal-like (hormone receptor negative and Her-2 negative) [3]. The molecular typing of breast cancer is a huge breakthrough in cancer theranostics which not only helps to predict the prognosis of patients, but also effectively guides the following treatment schedule [4–6]. Among the three breast cancer subtypes, Basal-like breast cancer has the poorest clinical outcome and the highest probability to reoccur than other two subtypes of cancers [7]. Apart from molecular typing, tumor DNA sequencing is also an important reference for treatment decision. Patients with germline mutations in *BRCA* might benefit from PARP inhibitor treatment while patients harboring alterations in *ERBB*-*2* or *ESR1* are more likely to develop resistance to standard therapies [8–13].

As a highly conservative signaling pathway, the retinal determination gene network (RDGN) was originally found to regulate *Drosophila* eye specification. Then, RDGN was reported to participate in the organ development in mammals [14]. At present, it has been well established that aberrantly expressed RDGN signals involve in the proliferation, apoptosis, stemness, and metastasis of cancer cells [15]. It has been known that RDGN comprises multiple members: dac/Dach (dominant suppressor of ellipse), eya/Eya (tyrosine phosphatase eyes absent), so/Six (Six family transcription factor sine oculis), as well as ey/toy (Pax6-like homeodomain proteins) [16]. In the main components of RGDN, DACH family generally plays a role as tumor suppressor while EYA and SIX families most likely act as oncogenes [17–21]. However, there are contrary reports on RDGN’s function in different cancers. For example, DACH1 protein levels were increased with the invasiveness of the ovarian cancer and subcellular distribution of DACH1 changed from nucleus in normal tissue to cytoplasm in cancer [22]. As a negative regulator of Wnt pathway, SIX3 inhibited breast cancer carcinogenesis and metastasis through recruiting the LSD1/NuRD complex [23]. In consistent with this experimental study, expression profile analysis indicated that high SIX3 mRNA level was a protective factor for OS and RFS of basal-like breast cancer patients [24]. Several studies proved that EYA4 behaved as a tumor suppressor and associated with favorite prognosis in hepatocellular carcinoma and pancreatic ductal adenocarcinoma [25, 26]. In summary, multiple studies suggested that members of RDGN family played distinct roles depending on the cancer type. Our previous studies showed that RDGN was dysregulated in tumors with a coordinated fashion: downregulated DACH1 in accompany with upregulated EYA1 and SIX1 in tumors [24, 27, 28].

Several groups attempted to address the prognostic and therapeutic response value of DACH1 in breast cancer. Machine learning methods such as Artificial Neural Networks have been utilized to identify biomarkers of breast cancer. Using Artificial Neural Networks approach, Powe et al. [29] found that DACH1 had a positive association with ER and exerted a strong influence on ER associated markers. Consisting with our study, nuclear DACH1 expression was observed in normal and Luminal breast cancer tissues. Patents with high expression of DACH1 demonstrated longer survival and disease-free interval as well as reduced metastasis risk [29]. However, prognostic value of DACH1 was not independent of clinical stage and Nottingham Prognostic Index [29]. Aromatase inhibitors (AI) are standard adjuvant treatment for postmenopausal luminal A subtype breast cancer [30]. However, resistance is still a major clinical problem for improving long term survival. Thomsen et al. [30] performed global gene expression analysis to measure gene expression profile of 23 ER positive breast cancer patients treated with adjuvant AI and collected follow-up information for relapse. Twenty-six genes including DACH1 were shown to exhibit altered expression in tumors from patients with relapse versus non-relapse. Ingenuity pathway analysis indicated DACH1 was linked with cyclin D1, cyclin A1, NRG family, and PLC1 [30]. It is interesting to mention that four methylation markers (RASGRF1, CPXM1, HOXA10, and DACH1) in circulating cell-free DNA could discriminate cancer from normal with high sensitivity (0.86) and specificity (0.83) in early breast cancer [31]. Among the 4 genes, RASGRF1 and CPXM1 represented common breast cancer marker, while HOXA10 and DACH1 represented luminal-dominant marker and triple negative dominant marker, respectively. CpG islands of those 4 genes were located in the promoter region and associated with H3K4Me3 enrichment. Those studies clearly indicated that the unbalanced RDGN status might be a useful biomarker to predict the prognosis of breast cancer patients. In this study, based on the results of bioinformatics analyses, we combined several members of RDGN and some well-accepted prognostic indicators to construct a predictive model for breast cancer patients.

## Methods

### Gene expression profiles

Two gene expression profiles (GSE25066 and GSE1456) were downloaded from Gene Expression Ominibus (https://www.ncbi.nlm.nih.gov/geo/). GSE25066 dataset was uploaded by Hatzis et al. [32] consisting of 508 breast cancer samples. GSE1456 dataset with 159 breast cancer samples was uploaded by Pawitan et al. [33]. Both GSE25066 and GSE1456 datasets were based on Affymetrix Human Genome U133A Array Platform. The pre-processed gene expression data of The Cancer Genome Atlas (TCGA) were obtained from UCSC Xena (https://xena.ucsc.edu/).

### Gene set enrichment analysis (GSEA)

GSEA software (version: 4.0.3) was utilized for enrichment analysis [34]. Gene set databases c2.cp.kegg.v7.0.symbols.gmt and c5.all.v7.0.symbols.gmt were used for KEGG pathways and GO-terms enrichment analyses. We set gene size > 15, False discovery rate < 0.25, P < 0.05, |NES| > 1 as positive criteria.

### Survival analysis

The survival analysis was conducted by online analysis tool Kaplan–Meier-plotter (https://kmplot.com/analysis/). Kaplan–Meier-plotter could assess the effect of over 54,000 genes on the prognosis of multiple cancers [35]. In this study, we used the overall survival (OS), relapse-free survival (RFS), distant metastasis-free survival (DMFS), and post-progression survival (PPS) as main parameters to evaluate to the influence of RDGN on the outcomes of breast cancer patients.

### Predictive model construction and verification

The three members of RDGN (DACH1, EYA2, and SIX1) and several well-established molecular biomarkers were included in Cox univariate regression analysis. According to the results of Cox univariate regression analysis, we selected RDGN members with statistical significance (P < 0.10) and other verified molecular biomarkers to construct Cox proportional hazards model. Then, we used time-dependent ROC curves to evaluate the accuracy and specificity of the risk model. Lastly, we used K-M plotter curves to assess the difference in prognosis between patients with high risk index (33.3 in the top percentile) and low risk index (33.3 in the last percentile). GSE1456 was the training set and GSE25066 was the validation set.

## Statistical analysis

The comparisons between different groups were conducted by Students’t test. All statistical results with a P value < 0.05 were considered significant. The survival curves were performed by Kaplan–Meier curves with log-rank test. Statistical analyses were conducted with Graphpad Prism 8.0 and R software (version 3.6.0 with package survminer and survivalROC).

## Results

### KEGG pathways and GO-terms enrichments

**Fig. 1:**
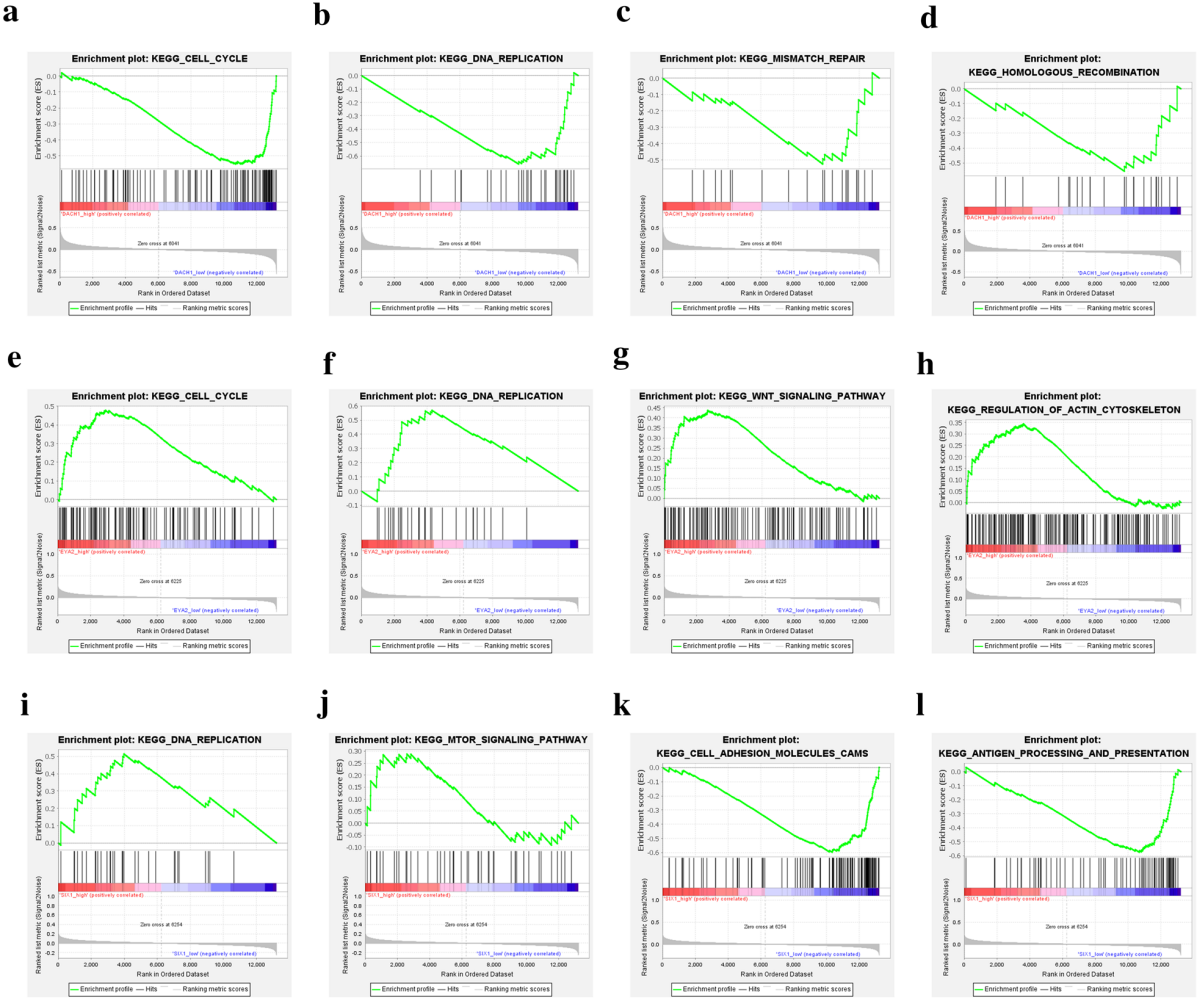
The results of GSEA (KEGG pathways). The gene profiles of samples with DACH1 low expression were significantly enriched in cell cycle (**a**), DNA replication (**b**), mismatch repair (**c**), and homologous recombination (**d**); The gene profiles of samples with EYA2 high expression were significantly enriched in cell cycle (**e**), DNA replication (**f**), Wnt signaling pathway (**g**), and regulation of actin cytoskeleton (**h**); The gene profiles of samples with SIX1 high expression were significantly enriched in DNA replication (**i**) and mTOR pathway (**j**); The gene profiles of samples with SIX1 low expression were significantly enriched in adhesion molecules (**k**) and antigen processing and presentation pathway (**l**)

**Fig. 2:**
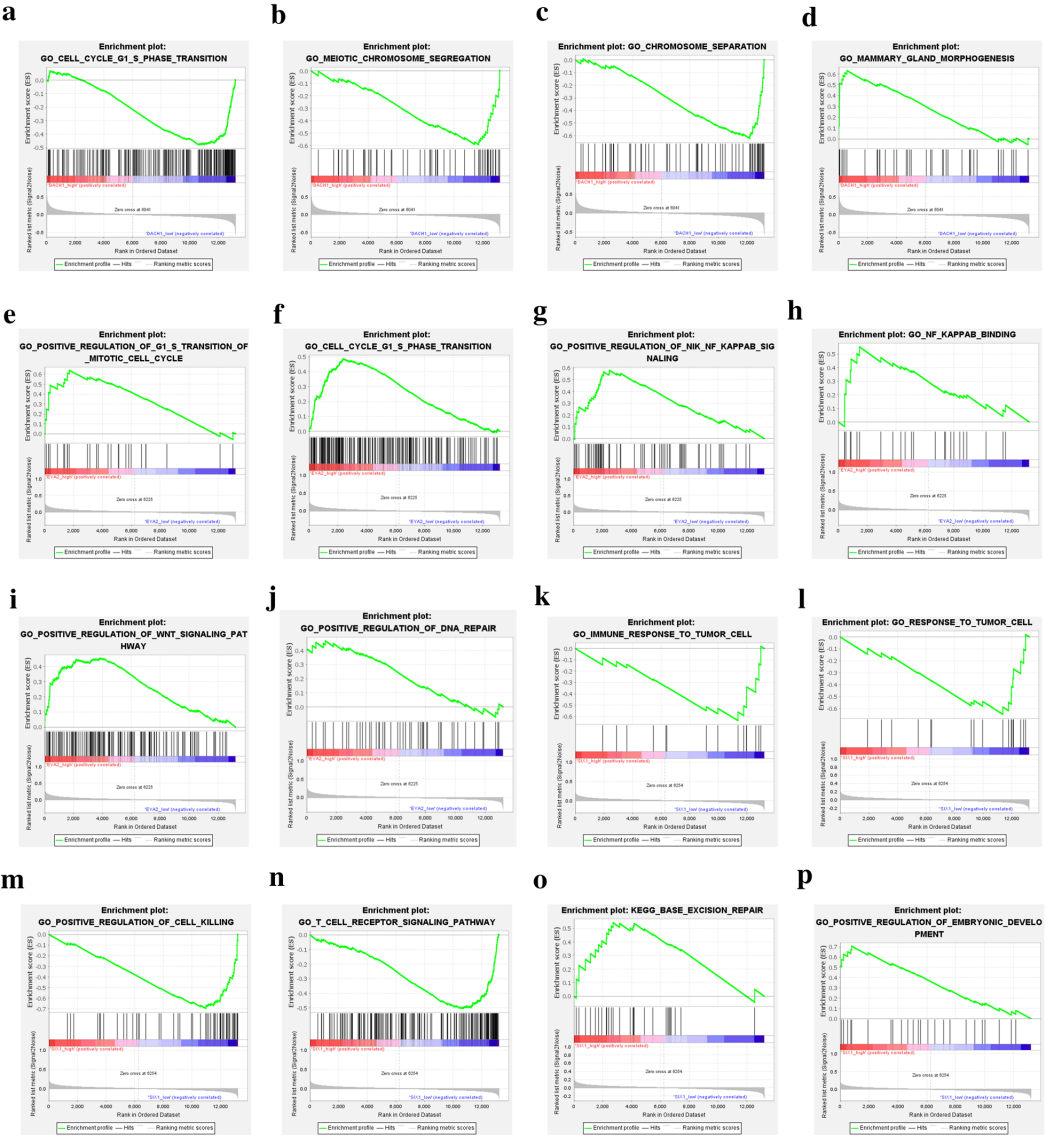
The results of GSEA (GO terms). The gene profiles of samples with DACH1 low expression were significantly enriched in cell cycle G1-S transition (**a**), meiotic chromosome separation (**b**) and chromosome separation (**c**); The gene profiles of samples with DACH1 high expression were significantly enriched in mammary gland morphogenesis (**d**); The gene profiles of samples with EYA2 high expression were significantly enriched in (positive regulation) cell cycle G1-S transition (**e** and **f**), (positive regulation) NF-κB signaling pathway (**g** and **h**); positive regulation of Wnt pathway (**i**) and positive regulation of DNA repair pathway (**j**); The gene profiles of samples with SIX1 low expression were significantly enriched in (immune) response to tumor (**k** and **l**), positive regulation of cell killing (**m**), and T cell receptor pathway (**n**); The gene profiles of samples with SIX1 high expression were significantly enriched in base excision repair (**o**) and embryonic development (**p**)

Based on dataset GSE25066, we separately analyzed the difference of gene profiles for high and low expression of DACH1, EYA2 and SIX1. The results of GSEA showed that DACH1 level was negatively related with cell cycle, DNA replication, mismatch repair, and homologous recombination pathways (Fig. 1a–d). The GO-terms enrichment demonstrated that DACH1 level was negatively correlated with cell cycle G1/S phase translation, meiotic chromosome segregation but positively related to mammary gland morphogenesis processes (Fig. 2a–d). On the contrary, EYA2 level was positively related with cell cycle, DNA replication, Wnt signaling pathway, regulation of action cytoskeleton pathways (Fig. 1e–h). Correspondingly, the GO-terms enrichment indicated that EYA2 was positively correlated to cell cycle G1/S phase translation, NF-κB pathway, Wnt pathway, DNA repair processes (Fig. 2e–j). Similar to EYA2, SIX1 level was positively correlated to DNA replication, mTOR pathway, cell adhesion molecules, and antigen presentation pathways (Fig. 1i–l). The results of SIX1 GO-terms enrichment analysis revealed that SIX1 was negatively related to anti-tumor immune response and T cell activity but positively correlated to embryonic development (Fig. 2k–p).

### Decreased DACH1 predicting poor prognosis of breast cancer

**Fig. 3:**
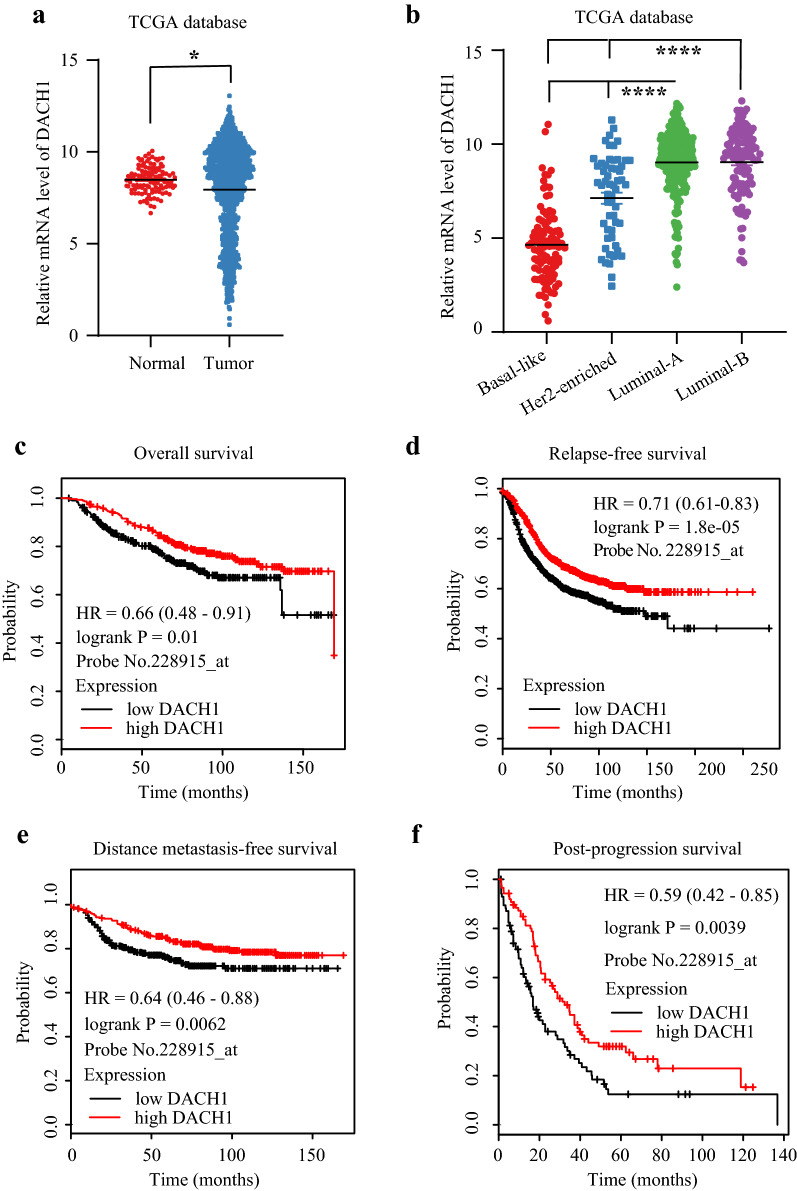
The relationship between DACH1 and breast cancer. **a** DACH1 is downregulated in breast cancer compared with normal tissues; **b** DACH1 level is higher in Luminal A and B subtypes than Basal-like and Her-2 enriched subtypes; Survival curves of DACH1 high or low breast cancer patients: overall survival (OS) (**c**), relapse-free survival (RFS) (**d**), distant metastasis-free survival (DMFS) (**e**), and post-progression survival (PPS) (**f**)

Based on the expression profiles from TCGA database, we compared the DACH1 level between normal breast tissues and breast cancer samples. The results showed that DACH1 level in tumor tissues was significantly lower than normal tissues (P < 0.05) (Fig. 3a). Further analysis showed that DACH1 was remarkably downregulated in Basal-like subtype compared with Luminal A or B or Her-2 subtypes (P < 0.0001) (Fig. 3b). The following survival analyses confirmed that high DACH1 level was a favorable factor for patients’ prognosis. Patients with higher DACH1 (above median level) had longer OS (HR = 0.66, P = 0.01) (Fig. 3c), RFS (HR = 0.71, P < 0.0001) (Fig. 3d), DMFS (HR = 0.64, P = 0.0062) (Fig. 3e), and PPS (HR = 0.59, P = 0.0039) (Fig. 3f).

### Increased EYA2 and SIX1 predicting poor prognosis of breast cancer

**Fig. 4:**
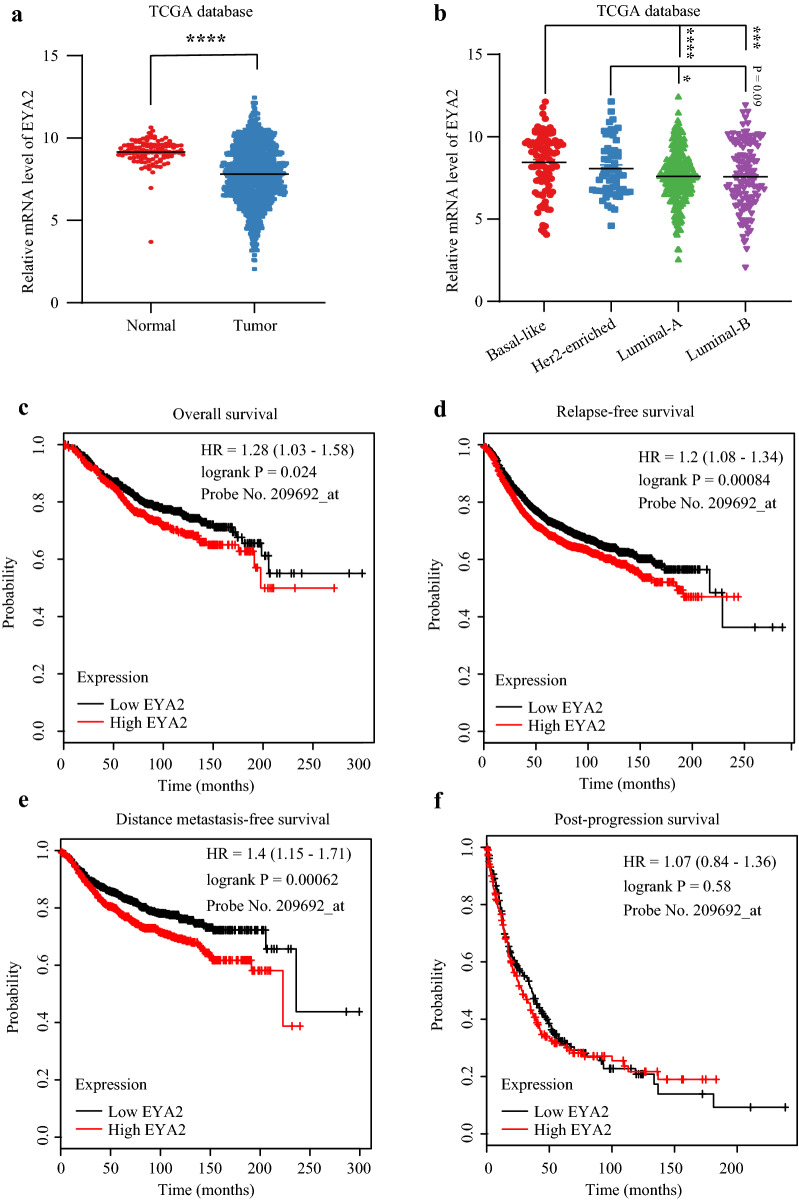
The relationship between EYA2 and breast cancer. **a** EYA2 is lower in breast cancer compared with normal tissues; **b** EYA2 level is lower in Luminal A and B subtypes than Basal-like and Her-2 enriched subtypes; Survival curves of EYA2 high or low breast cancer patients: overall survival (OS) (**c**), relapse-free survival (RFS) (**d**), distant metastasis-free survival (DMFS) (**e**), and post-progression survival (PPS) (**f**)

**Fig. 5:**
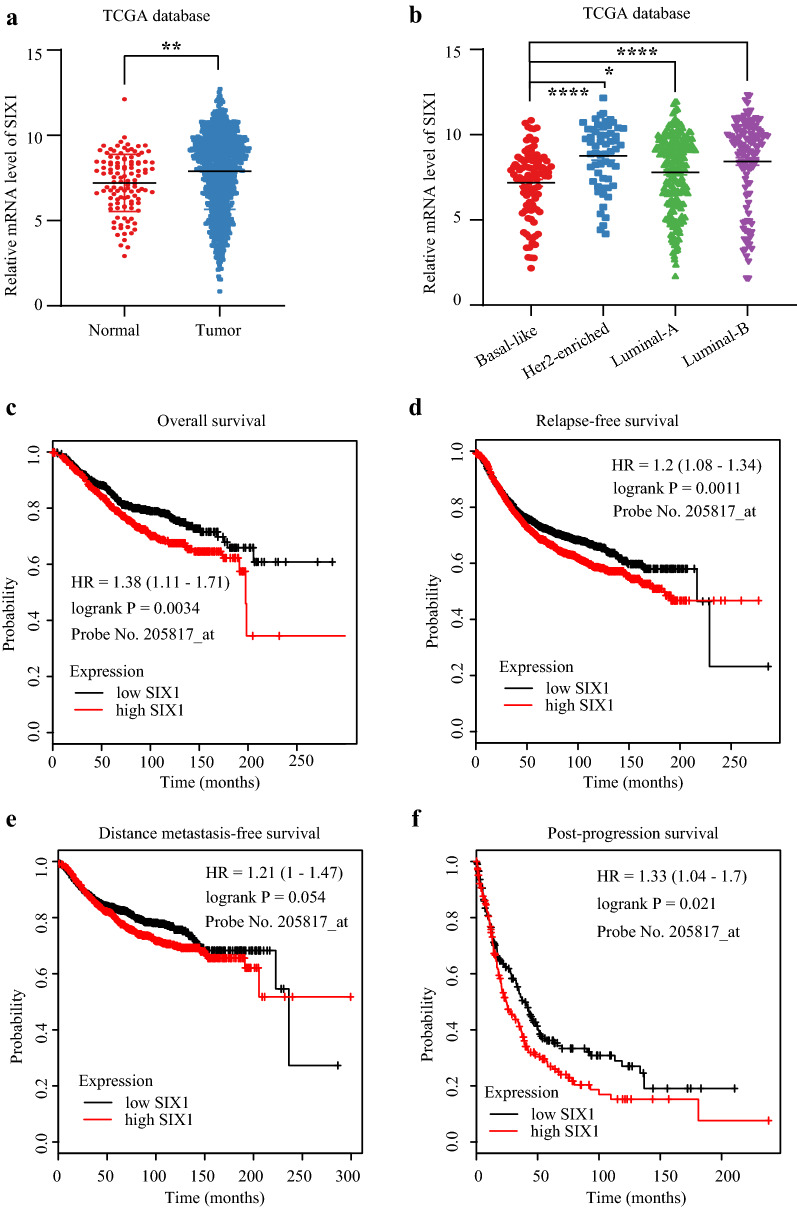
The relationship between SIX1 and breast cancer. **a** SIX1 is upregulated in breast cancer compared with normal tissues; **b** SIX1 level is higher in Luminal A, B, and Her-2 enriched subtypes than Basal-like subtype; Survival curves of SIX1 high or low breast cancer patients: overall survival (OS) (**c**), relapse-free survival (RFS) (**d**), distant metastasis-free survival (DMFS) (**e**), and post-progression survival (PPS) (**f**)

The expression data of EYA2 and SIX1 were extracted from TCGA database. EYA2 level was lower in cancer samples than normal breast samples while SIX1 level was higher in cancer samples than normal breast samples (Figs. 4a and 5a). Moreover, among all breast cancer subtypes, Basal-like subtype possessed the highest EYA2 level (Fig. 4b). Contrarily, the SIX1 level in Basal-like subtype was markedly lower than Luminal and Her2-enriched subtypes (Fig. 5b). Generally, both EYA2 and SIX1 were risk factors for prognosis of breast cancer patients. Patients with high EYA2 (above median value) had shorter OS (HR = 1.28, P = 0.024) (Fig. 4c), RFS (HR = 1.2, P = 0.00084) (Fig. 4d), DMFS (HR = 1.4, P = 0.00062) (Fig. 4e), but had no effect on PPS (HR = 1.07, P = 0.58) (Fig. 4f). Similarly, patients with higher SIX1 (above median value) had poorer OS (HR = 1.38, P = 0.0034) (Fig. 5c), RFS (HR = 1.2, P = 0.0011) (Fig. 5d), DMFS (HR = 1.21, P = 0.054) (Fig. 5e), as well as PPS (HR = 1.33, P = 0.021) (Fig. 5f).

### Predictive model construction and verification

**Fig. 6:**
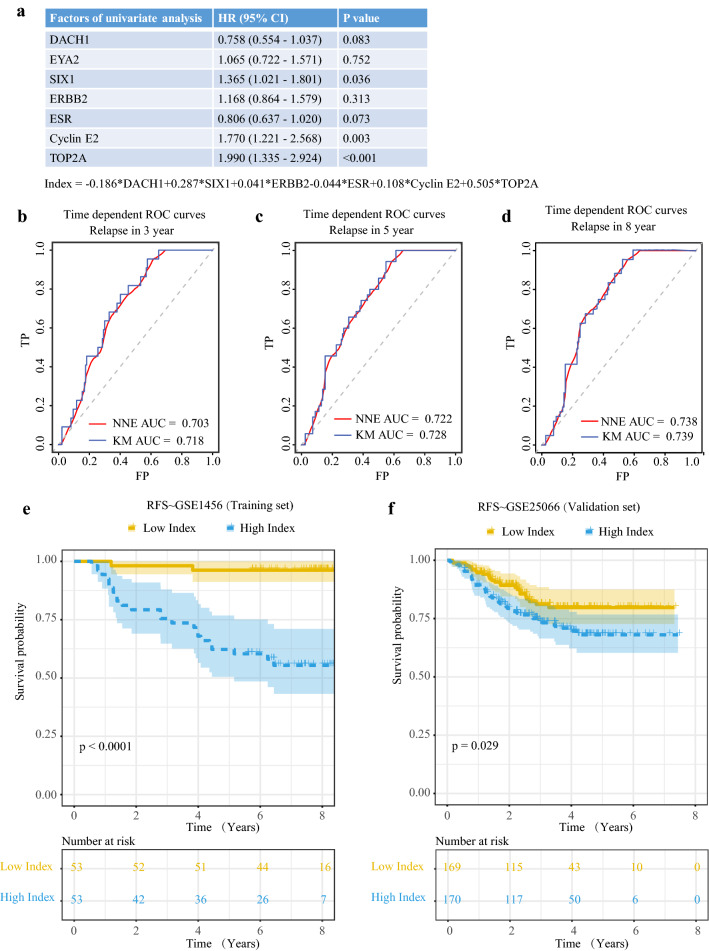
The construction and verification of predictive model. **a** The results of Cox univariate regression analysis; The time dependent ROC curves predicting the relapse within 3 (**b**), 5 (**c**), and 8 (**d**) years; (**e**) The K-M plotter curves of patients with high or low risk index in training set GSE1456; (**f**) The K-M plotter curves of patients with high or low risk index in training set GSE25066

According to the clinical information and gene matrix of GSE1456, we performed Cox univariate regression analysis to select prognosis indicators from RDGN. Eventually, we utilized DACH1 (HR = 0.758, P = 0.083), SIX1 (HR = 1.365, P = 0.036), as well as other previously verified molecular factors to construct a predictive model for potential relapse of breast cancer patients (Fig. 6a). Besides, we referred to the gene set designed by Pawitan et al. [33] and involved ERBB2, ESR, Cyclin E2, as well as TOP2A in our model to increase predictive power. By Cox proportional hazard model, we constructed the risk index = − 0.186*DACH1 + 0.287*SIX1 + 0.041*ERBB2 − 0.044*ESR1 (ESR) + 0.108*Cyclin E2 + 0.505*TOP2A. Then, the time-dependent ROC curves showed that this index could effectively distinct patients with high relapse risk within 3, 5, and 8 years (AUC > 0.7 in all conditions) (Fig. 6b–d). By K-M plotter method, we found that patients with high risk index exhibited shorter RFS in GSE1456 (P < 0.0001) (Fig. 6e). Subsequently, we utilized another dataset GSE25066 as verification. In GSE25066, patients with high risk index also had shorten RFS than individuals with low risk index (P = 0.029) (Fig. 6f).

## Discussion

RDGN is a highly conserved signal for proper organ development across taxa [36]. However, dysregulated RGDN expression is highly related to cancer initiation and progression. For breast cancer, decreased DACH1 led to accelerated cell cycle, enhanced stemness, invasion, and metastasis. In the previous study, we found that DACH1 regulated cell cycle via inhibiting Cyclin D1 [37] and reprogramed cell stemness by downregulating stemness-associated molecules such as SOX2, Nanog, as well as KLF4 [38]. Besides, it has been verified that DACH1 could suppress epithelial-mesenchymal transition (EMT), migration, metastasis by antagonizing YB-1-mediated transcriptional events, SNAI1-E-cadherin pathway, and IL-8 transcription in breast cancer cells [39–41]. Notably, it has been revealed that DACH1 was expressed in estrogen receptor breast cancer cells and acted as an endogenous inhibitor of estrogen signaling [42].

Contrary to DACH1, EYA2 facilitates proliferation, migration, invasion, and metastasis of breast cancer cells. EYA2 promoted breast cancer cellular proliferation and distant metastasis as the downstream of EGFR [28, 43]. Different from the other members of RDGN, EYA2 had a phosphatase activity. Inhibiting the phosphatase activity of EYA2 could suppress EYA2-mediated malignant biological behavior [44, 45]. Similar to EYA2, SIX1 also play a pro-tumor role. SIX1 facilitated tumor growth by enhancing Warburg effect. Besides, SIX1 promoted EMT, metastasis, and chemotherapy resistance in breast cancer cells [46–48]. Our previous meta-analysis showed that increased SIX1 was closely related to poor prognosis of breast cancer patients [24].

In the present study, we firstly explored the effects of DACH1, EYA2, and SIX1 on downstream signaling pathways. We found that DACH1 was negatively related to cell proliferation-associated pathways while EYA2 and SIX1 were positively correlated with cell cycle or DNA replication pathways. Moreover, increased EYA2 and SIX1 were highly related with some upregulated oncogenic pathways such as Wnt and NF-κB pathways. It was worth mentioning that SIX1 level was positively correlated to DNA repair activity which might contribute to chemotherapy resistance in breast cancer patients with high SIX1 expression [48]. The following survival analyses showed DACH1, EYA2, and SIX1 were predictive biomarkers of prognosis of breast cancer patients. Then, we constructed a RDGN-based framework which could effectively distinguish patients with high risk of relapse.

Actually, gene expression profiling has been utilized in clinical practice for treatment decision of early stage breast cancer patients with ER positive and lymph node metastasis negative [49, 50]. Based on the results of gene expression profiling, patients could be categorized into high risk, middle risk, and low risk groups. Patients belonging to high risk group might benefit from adjuvant chemotherapy while patients belonging to low risk group could avoid unnecessary treatment [51]. Up to now, several commercial gene expression profiling tests are available including Oncotype DX (21 genes assay), Prosigna (PAM 50), MammaPrint (70 genes assay), and EndoPredict [52–54]. These test panels are usually used after breast cancer surgery with known hormone receptor and lymph node metastasis statuses. As a typical example of precious medicine, gene expression profiling is a valuable aid to guide whether or not conduct subsequent chemotherapy. Among them, Oncotype DX is the most commonly used panel [55]. In the 21 genes of Oncotype DX, 16 genes were oncogenic pathway-associated genes and 5 genes were control genes [56]. According to the results of a prospective study (NCT00310180), 93.8% of lymph node negative, ER positive, and HER2 negative breast cancer patients with low Oncotype DX relapse scores were free of disease progression after 5 years follow-up [57]. Besides, a phase 3 trial SWOG-8814 indicated patients with high Oncotype DX relapse score could benefit from additional chemotherapy (HR = 0.59, P = 0.033) while patients with low relapse score had no improvement in survival (HR = 1.02, P = 0.97) [58]. It has been clear that patients with high risk scores benefit from additional chemotherapy. However, for predictive models such as Oncotype DX, it is still uncertain about whether patients with middle relapse scores could benefit from additional chemotherapy [59]. Our predictive model was constructed based on RDGN members which was different from commercially available predictive tests. Theoretically, this RDGN-based model would provide complementary prognostic information. This model might provide extra information for deciding personal therapeutic scheme when patients are diagnosed as middle relapse risk individuals by other commercial tests.

We hereby constructed a prognostic model to predict the outcomes of breast cancer patients. To the best of our knowledge, this is the first RDGN-based predictive model for the risk of breast cancer relapse. Despite the promising results, some questions still remain. Firstly, due to the absence of housekeeping genes, it is hard to set a cutoff value for other extended data sets and avoid the impact of sequencing batches. Besides, this predictive model was constructed and validated by public database and retrospective studies. This model may need to be further validated in a randomized controlled trial. Most importantly, it is unclear that this predictive model could keep its validity on other sequencing platforms. Further studies are required to resolve the problem properly.

## Conclusion

Dysregulated RDGN is well known for breast cancers. Downregulated DACH1 and upregulated EYA2 and SIX1 were highly related to DNA replication and cell cycle pathways. Besides, decreased DACH1 and increased EYA2 and SIX1 heralded the poor prognosis of breast cancer patients. Combining RDGN members and other molecular biomarkers, we constructed a predictive model for the potential relapse risk. This RDGN-based model exhibited high accuracy and specificity to distinguish patients with high risk of relapse. The results of repeatability test in a verification set showed this model is stable. Generally, we believed that this model would be an important complement for commercially available predictive tests.

## Data Availability

GEO datasets involved in this study could be downloaded from https://www.ncbi.nlm.nih.gov/geo/. TCGA data are available at UCSC Xena (https://xena.ucsc.edu/).

## Abbreviations

RDGN: Retinal determination gene network
GEO: Gene Expression Ominibus
GSEA: Gene Set Expression Analysis
TCGA: The Cancer Genome Atlas
OS: Overall survival
RFS: Relapse-free survival
DMFS: Distant metastasis-free survival
PPS: Post-progression survival
EMT: Epithelial-mesenchymal transition
